# Lack of Association of White Matter Lesions with Ipsilateral Carotid Artery Stenosis

**DOI:** 10.1159/000336762

**Published:** 2012-03-14

**Authors:** Gillian M. Potter, Fergus N. Doubal, Caroline A. Jackson, Cathie L.M. Sudlow, Martin S. Dennis, Joanna M. Wardlaw

**Affiliations:** ^a^Division of Clinical Neurosciences, Brain Research Imaging Centre, University of Edinburgh, Western General Hospital, Edinburgh, UK; ^b^SINAPSE Collaboration, SFC Brain Research Imaging Centre, Western General Hospital, Edinburgh, UK

**Keywords:** Leukoaraiosis, White matter lesions, Aetiology, Carotid stenosis, Atheroma, Thromboembolism, Magnetic resonance imaging

## Abstract

**Background:**

White matter lesions (WML) are commonly seen on brain MRI and are generally considered a marker of tissue damage from cerebral small vessel disease. WML are associated with increasing age and vascular risk factors, but their precise cause is unknown. A role for carotid artery atherothromboemboli has been suggested. If this is the case, more WML would be expected ipsilateral to increasing degrees of carotid stenosis.

**Methods:**

We recruited patients with ischaemic stroke from two large, separate prospective stroke studies, assessed with brain MRI and carotid Doppler ultrasound. We scored hemispheric WML visually in periventricular and deep locations. We assessed the association between carotid stenosis asymmetry and WML asymmetry, and vice versa. Further, we assessed the association between carotid stenosis and ipsilateral WML, before and after adjusting for vascular risk factors, and tested associations between ipsilateral and contralateral stenoses and WML.

**Results:**

We recruited 247 (Study 1) and 253 (Study 2) patients. In Study 1 and Study 2, 36 (15%) and 29 (11%) patients had ≥50% carotid stenosis, and 27 (11%) and 15 (6%) had ≥70% stenosis, respectively. Carotid stenosis was asymmetric in 28 (11%) and 26 (10%) patients and WML were asymmetric in 22 (9%) and 11 (4%) patients in Study 1 and Study 2, respectively. We found no association between carotid stenosis and ipsilateral WML score, before or after adjusting for vascular risk factors or sidedness, but WML were strongly associated with increasing age (p < 0.001).

**Conclusion:**

In two large cohorts of ischaemic stroke patients, we found no association between carotid stenosis and ipsi- or contralateral WML. There is now substantial evidence that atherothromboemboli are unlikely to cause most WML or other forms of cerebral small vessel lesions. Future studies should focus on determining what causes the intrinsic small vessel pathological changes that appear to underlie most WML.

## Introduction

White matter lesions (WML), also termed ‘leukoaraiosis’ [1], are commonly seen on brain magnetic resonance imaging (MRI) in older people and are associated with symptoms common in old age, including impaired balance and gait, depression, cognitive impairment and dementia [2,3], as well as with worse functional outcome after stroke [4]. Their precise cause is unknown [5], but as well as being associated with increasing age, they are associated with hypertension, diabetes, other vascular risk factors [6] and markers of carotid atheroma such as intima media thickening and carotid plaques [7]. If WML are caused by emboli (either from carotid atheroma, cardiac or other sources), then they should be greater in the cerebral hemisphere ipsilateral to a more severe carotid stenosis. Here, we assessed the association between carotid stenosis and ipsilateral WML.

## Methods

We recruited patients who underwent brain MRI from two prospective studies: a hospital-based stroke register of consecutive patients with stroke or transient ischaemic attack presenting to a large academic teaching hospital between 2002 and 2005 (Study 1), and a study of patients with mild stroke presenting to the same hospital between 2005 and 2007 (Study 2). The recruitment periods did not overlap. Full details have been published previously [8,9], together with MRI findings. The patients were assessed by experienced stroke physicians, who recorded baseline demographics, National Institutes of Health Stroke Scale (NIHSS) scores, vascular risk factors and other details [8,9]. Hypertension and diabetes mellitus were defined as previous diagnosis of, or on current treatment for, hypertension or diabetes, respectively. Patients were assigned an Oxfordshire Community Stroke Project (OCSP) stroke subtype [10], modified following radiological assessment to a ‘final’ stroke subtype. Study 1 included all OCSP ischaemic stroke subtypes, and Study 2 included lacunar, partial anterior and posterior circulation stroke syndromes only. We performed routine stroke investigations on all patients (details published previously [8,9]). Written informed consent was obtained from all patients, and both studies were approved by the local Research Ethics Committee.

We performed carotid Doppler ultrasound, blinded to other imaging results and to most clinical features except that the patient had had stroke-like symptoms, using a 7.5-MHz linear transducer and optimized colour Doppler mode. Stenosis was defined according to the North American Symptomatic Carotid Endarterectomy Trial (NASCET) criteria [11] using peak systolic velocity measurements as previously described [12,13,14]. The carotid artery contralateral to the side of the body affected by the stroke symptoms was defined as symptomatic.

The patients underwent brain MRI (GE Signa 1.5T scanner), including axial diffusion- and T_2_-weighted imaging (T2WI), fluid-attenuated inversion recovery (FLAIR), gradient echo and T_1_-weighted sagittal imaging [8,9]. All patients in Study 2 and about a quarter of patients in Study 1 had MRI.

We defined recent infarcts as hyperintense on diffusion-weighted imaging, hypointense on the apparent diffusion coefficient map and either normal or hyperintense to the brain on FLAIR/T2WI. A neuroradiologist, aware of the side of acute stroke symptoms but blinded to other clinical and imaging data, assessed the brain MRI scans at non-overlapping time points (G.M.P. Study 1, J.M.W. Study 2). The reader of Study 1 was unaware of the hypothesis being tested and blinded to data from Study 2. Hemispheric WML were rated using FLAIR and/or T2WI using the Fazekas scale [15], scoring 0–3 (for deep and periventricular WML, where 0 = none and 3 = severe). For total hemispheric WML, we added scores in the deep and periventricular regions and obtained the average. Both neuroradiologists had undergone extensive WML rating training and rated a validated test set of 20 MRI scans for WML to standardise performance.

### Statistical Analysis

We examined both studies to identify if the data were sufficiently comparable before combining them for some of the analyses. Keeping the studies separate, we first sought evidence of WML score asymmetry between the cerebral hemispheres in individual patients and compared it with carotid stenosis asymmetry. We defined stenosis asymmetry as one carotid artery measuring <50%, and the contralateral artery measuring ≥50% stenosis, and WML asymmetry as a one-point difference in the Fazekas score between hemispheres, for both periventricular and deep locations. We combined the studies and assessed asymmetry of stenosis/WML and the association between carotid stenosis and ipsilateral WML before and after adjusting for potential confounders (age, diabetes and hypertension), using binary logistic regression. We analysed WML/carotid stenosis associations by comparing symptomatic versus asymptomatic, and left versus right, hemispheres within each patient. In the logistic regression model, we dichotomised the scores for overall (periventricular plus deep) WML as mild (0, 0.5, 1.0) versus severe (1.5, 2.0, 2.5, 3.0). For the analyses, we used Minitab Statistical Software (Version 15, Minitab Inc., State College, Pa., USA).

## Results

Study 1 included 247 (mean age 69 ± 13 years) and Study 2 included 253 (mean age 68 ± 11 years) acute stroke patients. The proportions of patients with vascular risk factors and the distribution of stroke severities were similar in both studies (table 1). There were 80 (32%) patients in Study 1 and 129 (51%) in Study 2 with a final diagnosis (taking account of clinical and imaging features) of lacunar stroke. The median periventricular and deep WML scores were 1 (IQR 1–2) in both studies. Carotid stenosis on at least one side of ≥50% was present in 36 (15%) and of ≥70% (including occluded) in 27 (11%) patients in Study 1, and 29 (11%) and 15 (6%) patients in Study 2, respectively. The range of stenosis in both studies ranged from 0 to 100% (occluded).

Carotid stenosis was asymmetric in 28 (11%) and 26 (10%) patients in Study 1 and Study 2, respectively (table 2); across both studies, only 4 patients had asymmetric WML (WML score higher distal to the stenosed side in 3, and distal to the non-stenosed side in 1). WML were asymmetric between hemispheres in 22 (9%) and 11 (4%) patients in Study 1 and Study 2, respectively; of these, across both studies, only 4 had asymmetric carotid stenosis (stenosis more severe proximal to the side with higher WML score in 3, and to the side with lower WML score in 1).

**Fig. 1:**
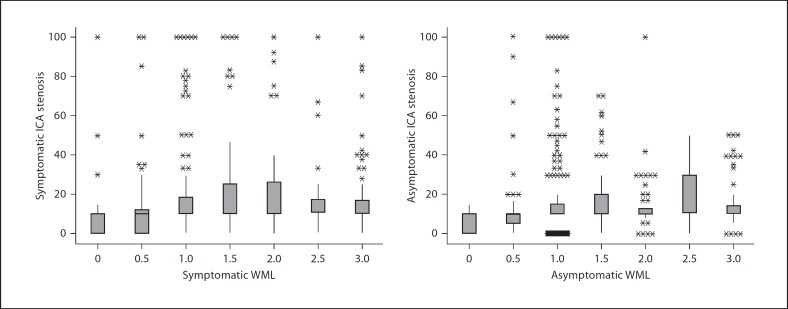
Relationship between hemispheric WML scores (Fazekas) and ipsilateral carotid stenosis (% NASCET) by symptomatic and asymptomatic sides, in both studies combined. WML scores were obtained by combining periventricular and deep WML scores and taking an average. Boxplots represent five-number summaries of NASCET stenosis (minimum, lower quartile, median, upper quartile and maximum values) with dots representing outliers. ICA = Internal carotid artery.

Combining both studies (1,000 carotid artery/hemisphere pairs), we found no association between carotid stenosis asymmetry and WML asymmetry (OR 1.15, 95% CI 0.39–3.41). In the two studies combined, there was no association between WML and ipsilateral carotid stenosis, whether symptomatic or not (fig. 1). We also found no association between WML and ipsilateral carotid stenosis after adjusting for potential confounders, whether considering carotid stenosis as symptomatic versus asymptomatic (p < 0.001) or as left versus right (p < 0.001) (table 3). Only increasing age was associated with the overall WML score.

## Discussion

In this study of two large cohorts of acute ischaemic stroke patients, we found no association between increasing carotid stenosis and increasing ipsilateral WML score, before or after adjusting for vascular risk factors. WML were strongly associated with increasing age (p < 0.001).

The main strengths of this study are the prospective collection of two cohorts of stroke patients who underwent brain MRI where we looked specifically at carotid stenosis and ipsilateral WML. Data collection was identical in each study using the same equipment and methods of imaging and quantifications. We included patients with a full range of carotid stenosis and WML, and applied several analyses, none of which demonstrated any association between carotid stenosis and WML.

The two neuroradiologists may have performed differently, introducing bias; however, both performed image reading to an internal standard, the studies had similar ranges of WML and our analysis initially examined the carotid stenosis/WML score within each study separately. We used visual WML scores rather than lesion volumes but, while volumes can give a more sensitive measure of WML, they may be distorted by accidental inclusion of infarcts that have similar signal to WML [16]. Although possibly less sensitive, visual WML scores are more specific as they do not suffer from this and related problems due to artifacts, and scores and volumes are closely related [17]. We made no adjustment for cardiac or aortic arch sources of the emboli, but this is unlikely to have had any significant influence on our results. The inclusion of some patients with either high-grade stenosis or carotid artery occlusion may have acted as a confounding factor due to the possibility of hypoperfusion-related WML [18], but if hypoperfusion were a mechanism, then we should still have found an association between WML and increasing stenosis. As relatively few patients had asymmetric stenosis (11% in Study 1 and 10% in Study 2), we cannot fully exclude an association between WML and carotid stenosis, although the absence of any association between stenosis and ipsilateral WML when the two studies were combined effectively rules this out. Also, these frequencies of asymmetry for carotid stenosis are typical of stroke patients, and WML are generally symmetrical, as in the present study, so if an association between carotid stenosis and WML does exist, it is likely to be weak and indirect.

Carotid stenosis is associated with a high risk of ipsilateral cortical ischaemic stroke and transient ischaemic attack. Several studies suggest that carotid stenosis is infrequent in lacunar stroke such that it may be coincidental [19], despite which, emboli may still be regarded as an important cause of lacunar stroke and WML. Amongst 12 previous studies (n = 7,843) assessing carotid stenosis versus WML score, 2 (n = 2,118) found an association between increasing stenosis and increasing *total brain* (not *ipsilateral*) WML and 10 (n = 5,725) did not (table 4) [18,20,21,22,23,24,25,26,27,28,29]. The populations and research methods differed in these studies; only some adjusted for some risk factors or age, so it is possible that any association between stenosis and WML is actually due to co-association with a third factor, e.g. age or hypertension, which was not adjusted for. Only 3 studies assessed the relationship between increasing carotid stenosis and ipsilateral WML (n = 1,395) [26,27,28], but 2 used CT, which is less sensitive to WML than MRI [27,28], and the third was small [26]. Our results are in agreement with those of Herholz et al. [29], who assessed asymmetric WML versus asymmetric carotid stenosis, but in only 20 patients. Our findings are also similar to those of Altaf et al. [26], although these authors did not consider carotid artery/hemispheres in relation to symptoms. There are now 11 studies, including ours, totaling 6,225 patients, which have not shown any association between WML and ipsilateral carotid stenosis, including 4 which examined individual artery/hemisphere units; of these, ours is the only study to account for the opposite artery/hemisphere unit by considering left versus right, and symptomatic versus asymptomatic, sides. A recent study by Schulz et al. [30] (presented only in abstract form) showed WML were not associated with carotid stenosis, agreeing with our data that WML form independently of atherosclerotic disease; these authors also assessed WML asymmetry versus carotid stenosis asymmetry and found no association.

In conclusion, we found no association between increasing carotid stenosis and ipsilateral WML. Existing data provide substantial evidence that atherothromboemboli have little role in WML formation (or by association, with most lacunar ischaemic stroke), and that any suggestion of an association in previous studies between embolic sources and WML may simply have been due to a third co-associated mediating factor, such as age or hypertension. Future studies should focus on determining what causes the intrinsic small vessel pathological changes that appear to underlie most WML.

## Funding

C.L.M.S., C.A.J. and the Study 1 stroke research register were funded by the Wellcome Trust (grant number 063668, Clinician Scientist Award to C.L.M.S.) and the Binks Trust. Study 2 was funded by the Chief Scientist Office of the Scottish Executive (grant number 217 NTU R37933) and the Wellcome Trust (grant number 075611). J.M.W. was funded by the Scottish Funding Council SINAPSE Initiative (Scottish Imaging Network, A Platform for Scientific Excellence, www.sinapse.ac.uk). G.M.P. was funded by the National Health Service Lothian Research and Development Office and the Chief Scientist Office of the Scottish Executive. F.N.D. was funded by the Wellcome Trust (grant number 075611). Imaging was performed in the Scottish Funding Council Brain Imaging Research Centre at the University of Edinburgh, and in the Neuroradiology Department, Western General Hospital, Edinburgh, UK.

## Disclosure Statement

The authors have no conflicts of interest to disclose.

## Figures and Tables

**Table 1:** Baseline clinical and imaging characteristics showing no significant differences between acute ischaemic stroke patients in Study 1 and Study 2

	Study 1 (n = 247)	Study 2 (n = 253)
Demographics		
Mean age ± SD, years	69 ± 13	68 ± 11
Males	133 (54)	165 (65)
Previous stroke	45 (18)	23 (9)
Diabetes	25 (10)	36 (14)
Hypertension	131 (53)	154 (61)
Previous AF	40 (16)	22 (9)
Lacunar stroke subtype	80 (32)	129 (51)
Median NIHSS score (IQR)	1 (0–3)^a^	2 (2–3)
Imaging parameters		
Median PVL^b^ (IQR)	1 (1–2)	1 (1–2)
Median DWML^b^ (IQR)	1 (1–2)	1 (1–2)

Values are numbers of patients with percentages in parentheses unless otherwise indicated.

PVL = Periventricular white matter lesions; DWML = deep white matter lesions; AF = atrial fibrillation.

a n = 230.

b PVL and DWML rated according to the Fazekas scale.

**Table 2:** Association between carotid stenosis asymmetry and WML asymmetry

	WML asymmetric	WML symmetric	Total
Study 1			
Stenosis asymmetric	2^a^	26	28
Stenosis symmetric	20	199	219
	
Total	22	225	247

Study 2			
Stenosis asymmetric	2^b^	24	26
Stenosis symmetric	9	218	227
	
Total	11	242	253

a WML score higher distal to the stenosed side in both patients.

b WML score higher distal to the stenosed side in one patient, and WML score lower distal to stenosis in the other patient.

**Table 3:** Association between hemispheric WML and ipsilateral carotid artery stenosis adjusted for vascular risk factors

	Symptomatic hemisphere WML		Asymptomatic hemisphere WML	
	OR (95% CI)	p	OR (95% CI)	p
Increasing age	1.10 (1.07–1.12)	0.00	1.10 (1.08–1.12)	0.00
Diabetes	0.84 (0.46–1.55)	0.58	0.90 (0.49–1.67)	0.75
Hypertension	1.18 (0.78–1.79)	0.44	1.15 (0.76–1.75)	0.50
Carotid stenosis (%)	1.0 (1.0–1.01)	0.44	0.99 (0.98–1.00)	0.24

**Table 4:** Previous published studies investigating the association between carotid artery stenosis measured by Doppler ultrasound^1^ and WML

Reference	Patients n	Subjects	Type of imaging	WML rating method	WML location assessed	Findings	Association
**Right % carotid stenosis versus global WML**						
Bots et al. [7], 1993	111	Randomly selected patients aged 65–85 years from the Rotterdam Scan Study (prospective follow-up of people aged ≥55 years investigating incidence of chronic disabling diseases)	MRI	Visual rating scale	Periventricular, deep	No difference in the prevalence of minimal and moderate-to-severe stenosis^a^ (right carotid artery) between groups with/without WML	Negative

**Unilateral/highest % carotid stenosis versus global WML**						
Romero et al. [20], 2009	1,971	Framingham Offspring Cohort (prospective epidemiologic study of young adults)	MRI	Volumetric, semiautomated	Global; location not stated	WML volume related to stenosis ≥50% after adjustment for vascular risk factors (OR 2.35, 95% CI 1.08–5.13)	Positive

Manolio et al. [21], 1999	3,502	Cardiovascular Health Study (cross-sectional study of men and women aged ≥65 years)	MRI	Visual rating scale	Periventricular, subcortical	WML associated with increasing severity of stenosis^b^ (p = 0.19)	Negative

Lindgren et al. [22], 1994	77	Randomly selected patients aged ≥35 years with no history of focal brain lesions	MRI	Visual rating scale	Periventricular, deep	No relationship between WML and stenosis ≥50% (p = not significant)	Negative

Schmidt et al. [23], 1992	234	133 consecutive stroke patients and 101 normal volunteers	MRI	Visual rating scale [15]	Periventricular, deep	No relationship between WML and stenosis^c^ in multivariate analysis adjusted for vascular risk factors	Negative

Adachi et al. [24], 1997	323	Patients with cerebrovascular disease, neurological disease, diabetes, ischaemic heart disease or medical examination of the brain	MRI	Visual, quantitative	Periventricular	No relationship between severity of periventricular WML and stenosis^d^	Negative

Fazekas et al. [25], 1988	52	Volunteers in a prospective field study on the incidence of cerebrovascular risk factors	MRI	Visual description	Deep and subcortical	Higher-grade stenosis^e^ not detected in subjects with or without WML	Negative

Bogousslavsky et al. [18], 1987	31	Patients with leukoencephalopathy and ischaemic stroke versus age- and sex-matched controls	CT	Visual description	Periventricular, centrum semiovale	Patients with WML less often had ≥50% stenosis^f^ or occlusion compared to controls (p < 0.05); OR not given	Negative

**% carotid stenosis versus ipsilateral hemispheric WML, each patient contributing two hemisphere-artery units**						
Altaf et al. [26], 2008	178	Recent anterior circulation TIA, minor strokes and amaurosis fugax, and minimum 30% stenosis	MRI	Volumetric, semiautomated	Periventricular, subcortical	WML volume not related to the degree of ipsilateral stenosis (p = 0.60)	Negative

Saba et al. [27], 2009	147	Consecutively registered patients aged ≥65 years undergoing CT of the brain and carotid arteries	CT	Visual rating scale	Hemisphere	Association between WML and carotid stenosis class, adjusted for age and vascular risk factors (OR 1.365, 95% CI 1.073–1.737; p ≤ 0.05)	Positive

Streifler et al. [28], 1995	1,197	Patients enrolled in NASCET with recent ischaemic symptoms and no cardiac source of embolism	CT	Visual rating scale	Periventricular	WML not related to the degree of ipsilateral stenosis^g^ [OR (severe versus mild stenosis) 1.08, 95% CI 0.73–1.62; p = 0.952]	Negative

**Carotid stenosis asymmetry versus hemispheric WML asymmetry**						
Herholz et al. [29], 1990	20	Patients evaluated because of suspected cerebrovascular disease	MRI	Visual rating scale	Hemisphere; location not stated	No correlation between hemispheric WML asymmetry and stenosis^1^ asymmetry (τ^b^ = 0.35; p = 0.074)	Negative

1 Except for Herholz et al. (stenosis measured by catheter angiography or Doppler ultrasound; stenosis graded 0 = no stenosis; 1 = <70% stenosis; 2 = ≥70% stenosis; 3 = occlusion).

a Graded normal: minimal = 1-15% stenosis moderate = 16-49% stenosis; severe = ≥50% stenosis.

b Graded as 0%, 1-24%, 25-49%, 50-74%, 75-99% and 100% stenosis.

c Graded 1-5: 0 = no atherosclerotic lesion; 1 = discrete atherosclerotic lesion at one side (<20%); 2 = 20-50% stenosis at one side or discrete atherosclerotic lesions at both sides; 3 = 50-70% stenosis at one side or 20-50% stenosis at both sides; 4 = >0% stenosis at one side, 50-70% stenosis at both sides, or occlusion at one side; 5 = ≥70% stenosis or occlusion at both sides.

d Graded 1-5: 1 = no lesions; 2 = <30% stenosis; 3 = 30-75% stenosis; 4 = ≥75% stenosis; 5 = occlusion; grouped as: 1 = unilateral grade 2 or lower; 2 = bilateral grade 2 or unilateral grade 3; 3 = bilateral grade 3 or unilateral grade 4; 4 = bilateral grade 4 or above or unilateral grade 5.

e Graded 0-3: 0 = no lesion; 1 = unilateral <20% stenosis; 2 = bilateral <20% or unilateral 20-50% stenosis; 3 = bilateral 20-50% or unilateral ≥50-70% stenosis.

f Graded as: normal or <50% stenosis; 50-74% stenosis; 75-99% stenosis; occluded.

g Graded as: mild = <30% stenosis; moderate = 30-69% stenosis; severe = 70-99% stenosis; occluded.

TIA = Transient ischemic attack; τb = tau beta statistic.
